# Endocrine Disrupting Chemicals Interfere With Leydig Cell Hormone Pathways During Testicular Descent in Idiopathic Cryptorchidism

**DOI:** 10.3389/fendo.2018.00786

**Published:** 2019-01-10

**Authors:** Patrick Fénichel, Nicolas Chevalier, Najiba Lahlou, Patrick Coquillard, Kathy Wagner-Mahler, Michel Pugeat, Patricia Panaïa-Ferrari, Françoise Brucker-Davis

**Affiliations:** ^1^Department of Reproductive Endocrinology, University Hospital of Nice, Nice, France; ^2^Institut National de la Recherche Médicale, UMR U1065, Université Nice-Sophia Antipolis, Nice, France; ^3^Department of Hormonology and Metabolic Disorders, Hôpital Cochin, APHP, Paris-Descartes University, Paris, France; ^4^Institut Sophia-Agrobiotech (INRA-CNRS, Nice University), Nice, France; ^5^Pediatric Endocrinology, Department of Pediatrics, CHU Lenval, Nice, France; ^6^Institut National de la Recherche Médicale, U1060 CaRMen, Fédération d'Endocrinologie, Hospices Civils de Lyon-1, Bron, France; ^7^Department of Biochemistry, University Hospital of Nice, Nice, France

**Keywords:** cryptorchidism, endocrine disrupting chemicals, testosterone, insulin like peptide 3, leydig cells

## Abstract

Cryptorchidism, a frequent genital malformation in male newborn, remains in most cases idiopathic. On the basis of experimental, epidemiological, and clinical data, it has been included in the testicular dysgenesis syndrome and believed to be influenced, together with genetic and anatomic factors, by maternal exposure to endocrine disrupting chemicals (EDCs). Here, we analyze how EDCs may interfere with the control of testicular descent, which is regulated by two Leydig cell hormones, testosterone, and insulin like peptide 3 (INSL3).

## Idiopathic Cryptorchidism

Undescended testis (UDT), also called cryptorchidism, is the most frequent congenital malformation in males, occurring in 2–8% of full-term male births (1–3). In young adults, it is associated with a higher risk for male infertility and testicular cancer (4, 5). Cryptorchidism cases can be characterized as unilateral or bilateral, transient (when spontaneous descent of the testis occurs within the first year of life) or persistent, and palpable or non-palpable according to the position of the undescended testis, following Scorer classification (6). With the exception of complex syndromes with multiple congenital abnormalities (7), most cases of UDT are unilateral, often transient and are considered as idiopathic (7). Idiopathic UDT is believed to be a multifactorial disease with anatomical, genetic and environmental risk factors (7–10). Anatomical factors could explain the frequent unilateral cases (10). Genetic causes such as mutations of INSL3, testosterone or their receptor genes (7, 11, 12) are rare in case of “idiopathic” UDT. Environmental factors, including *in utero* exposure to EDCs, have been proposed as co-factors for the occurrence of idiopathic UDT and other male reproductive developmental abnormalities (9). This environmental hypothesis is supported by: 1/ epidemiological studies showing, for example, temporal (13) or geographical differences (14), 2/ observations made in wildlife after environmental accidents, and 3/ experimental results in rodents, showing that exposure to several EDCs with estrogenic or anti-androgenic effects during fetal life, disturbs testicular descent (15). However, epidemiologic evidence in humans, remain scarce and the mechanisms which could link the EDCs exposure with UDT remain incompletely understood. In this review, we will analyze the data which support that EDCs with estrogenic or anti-androgenic effects may influence the occurrence of cryptorchidism, and how they may interfere with the hormonal control of testicular descent.

## Hormonal Control of Testicular Descent

Physiological descent of the testes during fetal development is quite well-understood, and has been described in several reviews (10, 16). Briefly, it includes two successive phases involving the participation of two ligaments: the cranial suspensory ligament (CSL) and the gubernaculum. The first phase, called the trans-abdominal phase, occurs in humans, between weeks 10 and 23. Due to the regression of the CSL and the growth of the gubernaculum (10), the testis migrates from the uro-genital ridge to the inguinal region. The second phase, inguino-scrotal, occurs after 28 weeks gestation. During this second phase, the regression of the gubernaculum will allow the testis to reach its definitive scrotal position. This will occur before birth in most cases, or during the neo-natal period for some of them (transient cryptorchidism). As supported by observations made in genetically modified rodents (17–19) or human genetic syndromes (7, 11, 12), the two-phases testicular descent is regulated by two testicular hormones: INSL3 and testosterone (18), which are produced by the differentiated Leydig cells (20). Classically, INSL3 is the regulator of the abdominal phase, and testosterone is necessary for the inguino-scrotal phase; but experimental data also support a role for INSL3 during the second phase, in association with androgens. INSL3 is a peptide hormone belonging to the relaxin family, specifically produced in the testis. Its receptor, RXFP2 (relaxin family peptide receptor 2), is developmentally expressed in the gubernaculum (21). Bilateral UDT and abnormal gubernaculum are present in INSL3 and RXFP2 knockout mice models (18, 19). Mutations of one of these two genes have been found in 4.7% of cryptorchid boys (7). The gubernaculum expresses the androgen receptor. Its regression is induced by androgens during the inguino-scrotal phase, as demonstrated by both animal models and human genetic syndromes (19–21). Impaired hypothalamic-pituitary axis leading to lack of testicular testosterone production or impaired androgen sensibility by lack of receptor expression, are associated with persistent, bilateral UDT, but they remain, like mutated INSL3/RFXP2 gene, very rare (17, 22, 23). Nevertheless, in the absence of mutations, impaired secretion of INSL3 and/or testosterone may influence testicular descent.

## Leydig Cell Hormones and Idiopathic Cryptorchidism

Two longitudinal case-control studies have tried to assess during the neonatal period, the Leydig cell hormones involved during testicular descent, in cryptorchidic boys. Bay et al. (24) were the first to report that INSL3 was decreased in idiopathic UDT (24). In their prospective study including 3 groups (control, Danish and Finnish cryptorchidic boys), they could first clearly establish the physiological ontology of testicular INSL3 secretion in boys. Levels were higher at birth and at 3 months, than in older pre-pubertal boys and significantly correlated to LH (24). They suggested that INSL3 is regulated at this period by the transient post-natal wave of gonadotropins. Secondly, they showed that INSL3 cord blood levels were reduced in persistent cryptorchidic boys and in the Finnish transient cryptorchidic subgroup when compared to controls. Thirdly, they observed that individual INSL3 levels in cryptorchidic boys increased significantly when assessed at birth and at 3 months for both transient and persistent cryptorchidism. However, at 3 months, they still observed a reduced level of INSL3 and an increased LH to INSL3 ratio in persistent cryptorchidic boys when compared to controls, while no more significant difference was noticed at that time in the transient group (24). Regarding the results of the persistent group, the authors suggested that in persistent cryptorchidism, Leydig cell dysfunction was already present in the perinatal period. As for the transient group, they suggested that the postnatal surge, which seems to physiologically stimulate INSL3 secretion, was able to normalize INSL3 secretion, contributing to the spontaneous testicular descent between birth and 3 months of a still normal testis (24).

**Figure 1:**
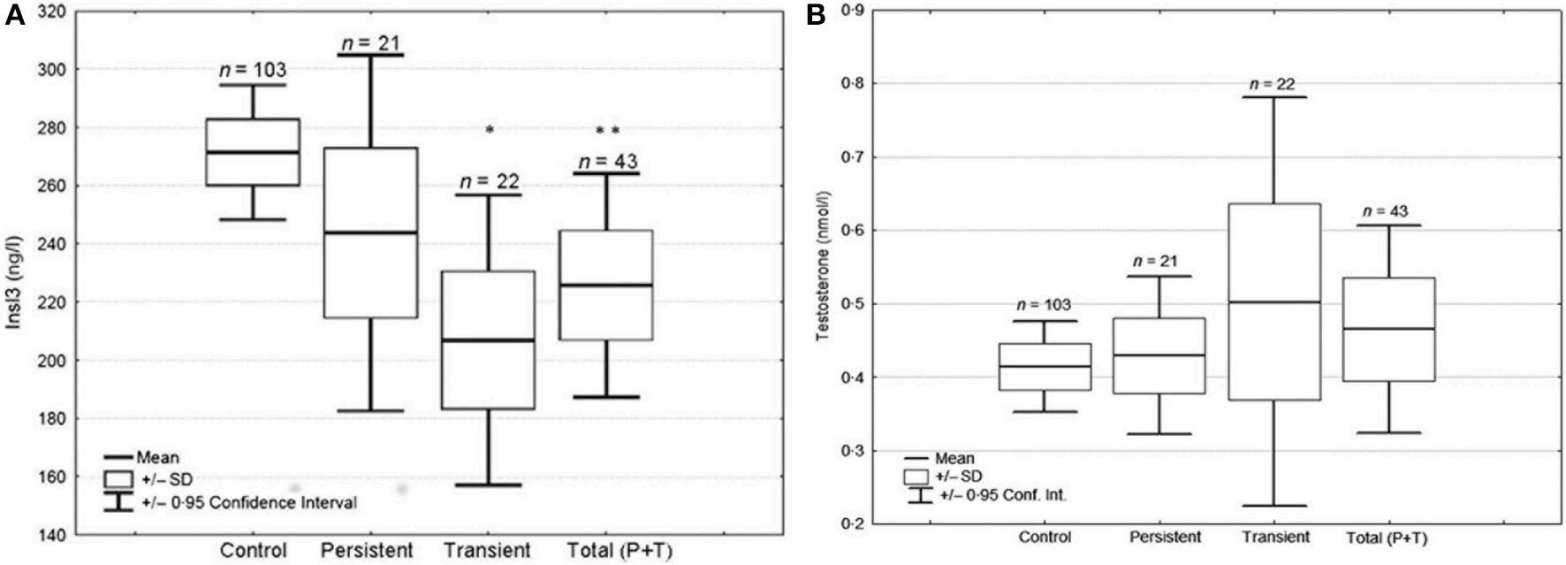
Testosterone **(A)** and INSL3 **(B)** cord blood levels in UDT and control groups. Boxand-whisker diagram of testosterone and INSL3 cord blood levels in controls, transient and persistent cases of cryptorchidism. The “total group” corresponds to the sum of transient and persistent cases. ^*^P = 0.029, ^**^P = 0.031 when compared to control group by logistic regression test. In Fénichel et al. (25).

From a prospective case-control study performed in Nice area (25), 180 boys born after 34 weeks of gestation, were assessed at birth and followed clinically during 1 year: 52 cryptorchid boys (48 unilateral, 4 bilateral; 26 transient, 26 persistent), and 128 controls matched for term, weight and time of birth. Cord blood INSL3 levels were significantly decreased in the total cryptorchidic group when compared to controls; this was mainly due to the transient cryptorchid subgroup, since the persistent group had values not significantly different from control (Figure 1). INSL3 was more significantly decreased in the group of 20 boys with non-palpable testes compared to the group of 21 with palpable testes, according to Scorer classification (25). In the whole population, INSL3 was positively correlated with LH and negatively with AMH, but with no other measured hormones.

Those two prospective studies on INSL3 in cryptorchidic boys confirm that neonatal INSL3 levels are decreased, but they seem to differ somewhat concerning the transient and the persistent cryptorchidic subgroups. However, when analyzed in details, they are rather concordant and complementary (25). Both teams found a relative large dispersion of the INSL3 values in all groups and the sizes of their subgroups were relatively small (24, 25). First, concerning the transient cases, the French team found a very significant decrease at birth and the Nordic team only in the Finnish subgroup (24); but the authors indicated that this subgroup had more severe (suprascrotal or worse) than mild (high scrotal) UDT, as opposed to the Danish subgroup, suggesting that this difference could explain the lower INSL3 levels. This hypothesis was confirmed later by the French study as reported above (25). One may now consider that transient cryptorchidism has a reduced secretion of INSL3 at birth, and that thanks to the postnatal LH wave (that correlates with it in both studies), it will be normalized at 3 months, contributing with normal Leydig cells, to the spontaneous testicular descent, as illustrated and proposed by Bay et al. (24). Second, regarding the persistent group, it was clearly associated in the Nordic study, with decreased INSL3 levels at birth still present at 3 months, in spite of the LH peak, while in the French study no significant INSL3 decrease was observed in cord blood (25). Tight analysis of the individual values showed (Figure 1) a wider dispersion suggesting a greater heterogeneity of the causal factors and/or of the degree of Leydig cell impairment.

Both studies found normal ranges of testosterone concentrations and LH/testosterone ratio in cryptorchidic newborn (25). The Nordic have reported an increased LH/testosterone ratio at 3 months of age (26, 27) or a decreased testosterone at 6 months, suggesting secondary Leydig cell dysfunction. Normal testosterone levels at birth contrast with lower INSL3 which appears at this time as a specific and sensitive marker of fetal Leydig cell impairment as proposed by Bay and Anand-Ivell (28). Fénichel et al. (25) reported in the cryptorchid group at birth, a positive correlation between LH and INSL3, but not between LH and testosterone. This suggest that INSL3, before and at birth, could be regulated by LH in a different way compared with testosterone as already proposed (20).

The reported INSL3 levels during neonatal period in cryptorchid boys triggers two related questions: 1/Is decreased cord blood INSL3 a cause or a consequence of UDT? 2/Could cord blood levels of INSL3 reflect what happened during fetal development and testicular descent? Classically, INSL3 is considered as regulating the first phase of testis migration during the second trimester of pregnancy. However, its contribution to the inguino-scrotal phase, has more recently been suggested. First, in the LH receptor knock-out mouse, testosterone administration causes an up-regulation of gubernaculum RXFP2 expression acting via the androgen receptor (29). Secondly, an INSL3 antagonist can inhibit the testosterone-induced inguinoscrotal descent (30). Last, as mentioned before, the higher levels of cord blood INSL3 in normal male newborns (24, 25) and the LH-dependent increase of INSL3 associated to spontaneous testicular descent in transient cryptorchidism, support a role for INSL3 during the inguino-scrotal phase (24). Moreover, there are several clues that support the INSL3 decrease in cryptorchidism as a causal factor, rather than a consequence. Briefly, as discussed in Fénichel et al. (25): “First, experimental induction of cryptorchidism in mice does not significantly alter the expression of INSL3 mRNAs in the testis (29). Secondly, testosterone, another Leydig hormone, was not affected in our cohort. Thirdly, if reduced cord blood INSL3 was a consequence of UDT, then the extent of the decrease might have been similar or even more marked in the persistent UDT boys, and this was not the case in our study (25).” This was in fact the case in the Nordic study for persistent cryptorchidism (24). One could integrate the different data in the following concept: persistent cryptorchidism is associated with low INSL3 levels already present at birth, persistent at 3 months, with a high LH/INSL3 ratio and altered testosterone or LH/testosterone ratio, suggesting impaired Leydig cells functioning as a consequence of UDT. In the transient forms of UDT that can be corrected after birth, lower reversible INSL3 levels suggest a functional causal effect with a down-regulation of INSL3 expression rather than a true testicular injury.

What could be the mechanism leading to fetal INSL3 decrease? Mutations or polymorphisms in the INSL3 and its receptor genes, in human patients with idiopathic UDT have been actively researched. Ferlin et al. (12) in a study involving 600 isolated cryptorchid infants, found only 1.1% of such mutations. On the other hand, INSL3 gene expression is negatively regulated by estrogens and positively by androgens, as shown in Leydig cells *in vitro* (30, 31). Thus, fetal exposure to estrogenic or anti-androgenic EDCs may be involved in the decrease of fetal INSL3 levels.

Moreover, the normal testosterone levels observed at birth in cryptorchidic boys, do not exclude an antagonistic action at the androgen receptor level mediated by an anti-androgenic EDC, which could indirectly impair the testosterone effect. Such an effect of antiandrogens on testicular descent has been demonstrated in animal models for several EDCs, like flutamide, vinclozolin, or phthalates.

What about human epidemiological data ?

## Idiopathic Cryptorchidism and Endocrine Disruptors

### Relationship Between Exposure to Endocrine Disruptors and Cryptorchidism

Maternal exposure to diethylstilbestrol (DES), a potent synthetic estrogen which was given to prevent miscarriages (32), has been associated with an increased risk of urogenital abnormalities in male newborn. Results from an American cohort estimated a doubling of the risk for cryptorchidism after *in utero* exposure to DES, with a higher risk when exposure occurred before week 11 of pregnancy (15). Several case-control studies have tried to link fetal exposure to EDCs and cryptorchidism, but prospective, longitudinal studies with a right methodology, are scarse. In a meta-analysis, Bonde et al. (33) could select 10 case-referent studies, addressing the risk of cryptorchidism following prenatal and post-natal exposure to endocrine disrupting chemicals. Summary Odds Ratio (OR) was not significantly increased. Only two studies (1, 34) and three risk estimates for beta-hexachlorocyclohexane (HCCB), p-p'- 1,1-Dichloro-2,2-bis(p-chlorophenyl) ethylene (DDE) and Polychlorinated Bisphenyls (PCBs) measured in maternal serum or milk, were significant. One of these prospective studies performed in Nice area (France) reported (1) an increased OR for PCBs (OR 2.74 [1.15, 6.53]) and for DDE concentrations (OR 2.16 [0.94, 4.98]) measured in maternal colostrum. More recently, a case-control study examined whether there was a link between maternal hair polybrominated diphenyl ether (PBDE) concentrations and the risk of UDT in male infants (35) and found that every 10-fold increase of the concentration of maternal hair BDE-99 or BDE-100, was associated with more than a doubling in the risk of UDT (35). Fernandez et al. (6), in a small cohort, correlated BPA and propyl-paraben concentrations in the placenta and the occurrence of hypospadias or cryptorchidism. Levels of two pesticides, heptachloroepoxide (HCE) and hexachlorobenzene (HCB), were found significantly higher in the fat taken during surgery for orchidopexy in a group of cryptorchid boys, when compared with controls (36). All these case-control prospective longitudinal studies report only indirect links; they are difficult to perform, have a limited sample size (37), are expensive, and usually assess only a small number of chemicals. As reviewed by Virtanen et al. (38), it is also hazardous to link UDT with a single chemical product. Fetus and newborn are exposed to many chemicals, which may present additive, antagonistic and/or synergistic effect. This was also shown *in vivo* in humans for UDT by Damgaard et al. (39), who found a correlation with a “cocktail” of several pesticides in breast milk, but not with any single pesticide, and by Brucker-Davis et al. (1) who built a score associating colostrum concentrations of DDE and several PCBs. In a study associating Danish and Finnish patients, seven PBDEs, all flame retardants, were detectable in milk and their sum was significantly higher in the group of Danish cryptorchid boys than in controls (40). Moreover, cord blood or maternal milk levels do not directly reflect fetal exposure during the window of testis descent. Amniotic fluid collected around 18 weeks of gestation has been proposed to evaluate INSL3 secretion at a time closer to this period (41). However, chemical concentrations are difficult to analyze, because they depend on varying dilution (41). Nevertheless, both epidemiological and experimental data, including those studying cryptorchidism, hypospadias and/or testicular cancer, support the hypothesis of a deleterious role for fetal exposure to EDCs.

How could EDCs disrupt testicular descent?

### Interference Between Exposure to Endocrine Disruptors and Leydig Cell Hormones

**Figure 2:**
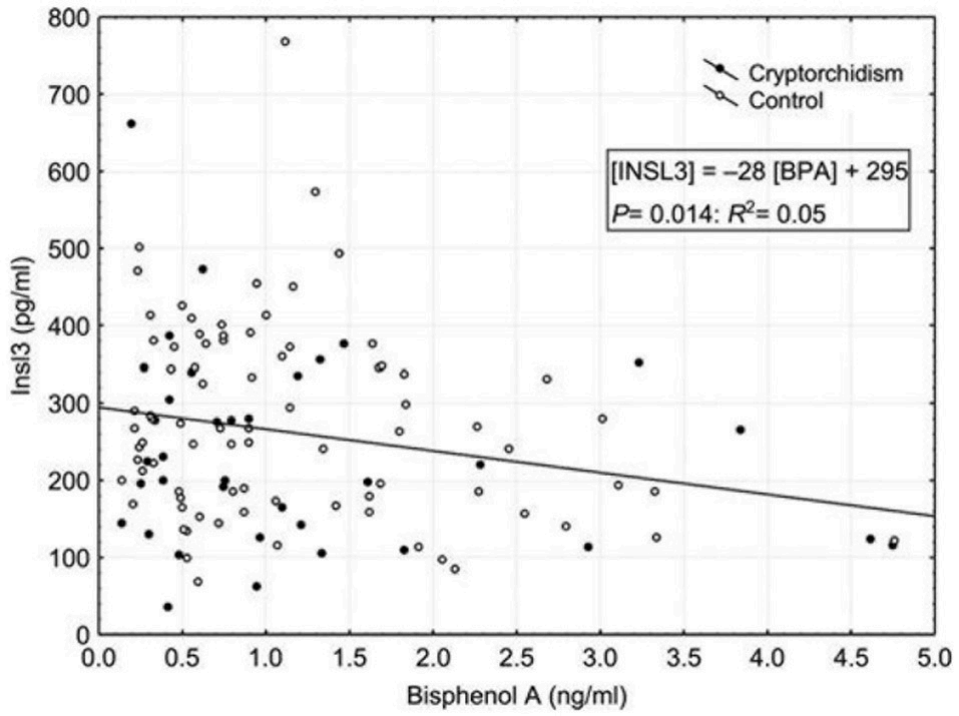
Insulin-like peptide 3 (INSL3) in relation to bisphenol A (BPA) in the whole study population of boys. Filled circles correspond to cryptorchid cases (*n* = 52), while open circles correspond to controls (*n* = 128). The linear regression is: Insl3 = −28 [BPA] + 295; *p* = 0.014, *R*^2^ = 0.05. In Chevalier et al. (43).

However, while it has been clearly shown that maternal exposure to estrogenic or anti-androgenic EDCs could induce cryptorchidism in rodents, it remains unproven that such environmental factors are operating in human idiopathic UDT, even if epidemiological studies with statistical correlations do exist as shown above (1, 6, 33–35). What could be the mechanism involved? Although cord blood levels of bisphenol A were not significantly increased in cryptorchidic boys (1.26 + 0.17 ng/ml vs. 1.14 + 0.13 ng/ml) when compared to control boys (42), when we looked for correlations between hormones and xenobiotics in the whole population (Figure 2) (43), we found a significant negative correlation between bisphenol A and INSL3 levels (*p* < 0.01). No significant correlation was found for testosterone or between both hormones and the other xenobiotics assessed (43). While the participation of BPA in this decrease remained small (*R*_2_ = 0.05), the statistical link was significant; this was consistent (negative effect at low dose) with the reported decrease of fetal INSL3 production observed by N'Tumba-Byn et al. (44) on human explanted fetal testes, cultured with low doses of BPA, even though these results were confirmed by Ben Maamar et al. (45) only in special culture conditions, omitting Human Chorionic Gonadotropin. From a mechanistic point of view, it is also in agreement with what is known from experimental data on the regulation of INSL3 gene expression and also on the disrupting effect of BPA. INSL3 gene expression is negatively regulated by estrogens, as shown in Leydig cells *in vitro* (31), and positively by androgens (46). In mice, maternal exposure to xenoestrogens, including the potent synthetic estrogen diethylstilbestrol (DES), results in down-regulation of INSL3 (but not testosterone) mRNA expression levels in Leydig cells (47, 48), and is associated with intra-abdominally located testes. In humans, an increased risk of cryptorchidism has been reported after fetal exposure to DES given as maternal treatment to prevent miscarriages (32). BPA, like DES, was initially designed as a synthetic estrogen, but it rapidly came to be widely used in the manufacture of plastics and epoxy resins. Because of its low affinity for the classical nuclear estrogen receptors ERα and ERβ (49), the classification of BPA as a xenoestrogen has also been debated (50). To explain BPA mechanisms of action, other receptors have been proposed, such as androgen receptor (51), estrogen related receptor gamma ERRγ (52) or membrane non-classical estrogen receptors (53–55). N'Tumba-Byn et al. (44), in reporting the negative effect of BPA on INSL3 Leydig cell secretion during human fetal testis culture, were able to exclude the ERα pathway by gene invalidation, and they suggested the participation of non-classical ERs (44). We have identified, in human testis, including Leydig cells, one of these membrane receptors, GPR30/GPER (G protein coupled estrogen receptor) for which BPA has a high affinity (53, 54). An anti-androgenic effect of BPA (51) has also been reported which could interfere with the positive regulation of testosterone on INSL3 gene expression (46). The lack of correlation between BPA and testosterone concentrations is not completely surprising since INSL3 and testosterone have been shown to be differentially regulated at the Leydig cell level. INSL3 secretion is dependent on the pituitary axis in a less acute way than testosterone (20) and synthesis of both hormones is also distinctly regulated (24, 35). Indeed, maternal BPA easily crosses the placenta (56, 57), and will be less easily conjugated and cleared by the fetus because of immature hepatic glucuronyl-transferase enzymes (58, 59) and active placental or fetal glucuronidases or sulfatases (58).

As in our previous report (41), there was no significant increase of BPA in boys with UDT when compared with controls (42). However, mean levels of BPA were higher in the cryptorchidic group, and strikingly more in the non-palpable vs. palpable subgroups, suggesting a link with the degree of migration defect. We have already reported a similar trend for INSL3 decreased levels (25). On the other hand, a single blood or spot urine BPA or conjugates test reflects short term exposure and not chronic exposure (60). Therefore, although exposure through diet is likely to be continuous, it cannot be concluded from this study, performed at the time of delivery, whether chronic fetal exposure to maternal BPA could disturb testicular descent at the time when INSL3 is most likely to be acting directly on the testis, in the first phase of testicular descent (gestational week 12–16). However, our data support the hypothesis that INSL3 is a target for endocrine disruption. Anand-Ivell and Ivell (41, 61) have even proposed that INSL3 could be a “monitor of endocrine disruption.” Indeed, INSL3 could be influenced by fetal exposure to several estrogenic and/or anti-androgenic EDCs acting as a “cocktail,” as suggested by epidemiological studies in idiopathic UDT (1, 37, 38).

Beside BPA, phthalates are among the strongest candidates for affecting the testis (62). There are robust data in rodents (23) and more recently in humans (63) supporting the deleterious effects of phthalates on testicular descent (23) and function (63). They may act on INSL3 gene expression/ action, on steroid hormone production or as an androgen antagonist (23). Effects of phthalates on INSL3 are sometimes contradictory, with some data showing an impact (62, 64), and others not (65). This discordance is likely due to a differential effect according to time of exposure or species (62, 64, 65).

In order to approach fetal exposure during specific windows of development, the assessment of phthalates in amniotic fluid has also been recently proposed with, however, the well-known technical difficulties associated with such studies (60, 61). Phthalates are able to interfere with the androgenic function of Leydig cells like DDE or PBDE (66) which have been both associated with cryptorchidism (1, 18/1, 34). This impairment of the androgenic action by phthalates may be involved in the experimental or epidemiological link reported with UDT (1, 35, 62), though the molecular mechanisms remain still largely unclear. but it is more difficult to demonstrate directly an antagonistic effect than a decreased peripheral blood level.

Acetaminophen (Paracetamol^*^) given to pregnant women has been suspected to increase the risk for male fetus to develop cryptorchidism (67, 68). In a xenograft model, it has been shown that prolonged exposure to acetaminophen reduces testosterone production by the human fetal testis (69). In another model of *ex vitro* culture of human fetal testis, exposure to acetaminophen was able to decrease INSL3 (but not testosterone) production during the critical window of the first abdomino-inguinal phase, (70), this could represent the mechanism by which this analgesic drug increases cryptorchidism risk.

## Conclusion

To conclude, experimental and epidemiological studies support the hypothesis of a deleterious role for fetal exposure to a cocktail of endocrine disruptors during the testicular descent; those compounds, acting as xenoestrogens and/or antiandrogens, may disrupt the secretion and/or action of INSL3 and testosterone, the two Leydig cell hormones, regulating testis descent, and lead to cryprorchidism in case of a genetic susceptibility context as recently suggested by Barthold and Ivell (71). However, direct evidence to support such a pathophysiological link explaining idiopathic UDT, remain scares. More prospective, longitudinal epidemiological studies and experimental models are necessary, exploring a more complete cocktail of common EDCs with possible estrogenic and/or anti-androgenic effects.

## Author Contributions

PF conceived and wrote the paper. NL preformed INSL3 and testosterone assay and discussed the results. NC participated to the discussion. PC made the statistical study. MP performed bisphenol assay. PP-F performed hormonal assays. KW-M supervised the clinical studies. FB-D directed the prospective study, discussed the results and participated to the writing of the paper.

### Conflict of Interest Statement

The authors declare that the research was conducted in the absence of any commercial or financial relationships that could be construed as a potential conflict of interest.
